# The Role of the Mammalian Target of Rapamycin (mTOR) in Pulmonary Fibrosis

**DOI:** 10.3390/ijms19030778

**Published:** 2018-03-08

**Authors:** Jessica Lawrence, Richard Nho

**Affiliations:** 1 Department of Veterinary Clinical Sciences, College of Veterinary Medicine & Masonic Cancer Center, University of Minnesota, St. Paul, MN 55108, USA; jlawrenc@umn.edu; 2 Division of Pulmonary, Allergy, Critical Care, and Sleep Medicine, Department of Medicine, University of Minnesota, 420 Delaware SE, Minneapolis, MN 55455, USA

**Keywords:** fibrosis, mammalian target of rapamycin (mTOR), idiopathic pulmonary fibrosis (IPF), radiation-induced pulmonary fibrosis (RIPF), phosphoinositide 3-kinase (PI3K), protein kinase B (AKT)

## Abstract

The phosphoinositide 3-kinase (PI3K)/protein kinase B (AKT)/mammalian target of rapamycin (mTOR)-dependent pathway is one of the most integral pathways linked to cell metabolism, proliferation, differentiation, and survival. This pathway is dysregulated in a variety of diseases, including neoplasia, immune-mediated diseases, and fibroproliferative diseases such as pulmonary fibrosis. The mTOR kinase is frequently referred to as the master regulator of this pathway. Alterations in mTOR signaling are closely associated with dysregulation of autophagy, inflammation, and cell growth and survival, leading to the development of lung fibrosis. Inhibitors of mTOR have been widely studied in cancer therapy, as they may sensitize cancer cells to radiation therapy. Studies also suggest that mTOR inhibitors are promising modulators of fibroproliferative diseases such as idiopathic pulmonary fibrosis (IPF) and radiation-induced pulmonary fibrosis (RIPF). Therefore, mTOR represents an attractive and unique therapeutic target in pulmonary fibrosis. In this review, we discuss the pathological role of mTOR kinase in pulmonary fibrosis and examine how mTOR inhibitors may mitigate fibrotic progression.

## 1. Introduction

Fibroproliferative disease refers to diseased states characterized by the overabundance of newly formed fibrous tissue produced by connective tissue cells. It is estimated that 45% of deaths in the United States are secondary to fibroproliferative disease [1,2]. While atherosclerosis is the most commonly recognized fibroproliferative disease, less common fibrotic diseases such as idiopathic pulmonary fibrosis (IPF) and radiation-induced fibrosis are also associated with high morbidity and mortality [2]. Unlike in normal wound healing, fibrotic cells proliferate long after an initial causative factor and often create an insidious disease process. Lung is a vital organ required for normal gas exchange, and its normal physiologic function depends on the presence of a thin alveolar membrane. The continued deposition of collagen-containing extracellular matrix (ECM) around alveolar not only impairs gas exchange but can also collapse the normal alveoli and replace underlying parenchymal cells. Lungs exposed to various endogenous and exogenous stimuli that induce injury can therefore lead to fibroproliferative disease if aberrant wound healing results in excessive fibroblast proliferation and matrix production. The causative mechanisms that induce fibroproliferative disease within the lung may often be diverse, however these diseases share a common pathogenesis of uncontrolled and progressive fibrosis that negatively impact lung compliance and gas exchange, which can ultimately lead to respiratory compromise. Idiopathic pulmonary fibrosis (IPF) and radiation-induced pulmonary fibrosis (RIPF) are both characterized by slowly progressive fibrotic foci that involve inappropriately activated (myo)fibroblasts that are responsible for encouraging fibrosis [3]. Pulmonary fibrosis represents a significant challenge and there are no effective cures for fibrosis, highlighting that the lack of effective therapy represents a significant unmet clinical need [2,3]. One challenge in determining effective treatment relates to the fact that fibrosis is typically a chronic, prolonged disease process and there is an incomplete understanding of underlying pathological mechanisms. Recent evidence has suggested that mammalian target of rapamycin (mTOR)-dependent pathways may play an integral role in promoting both IPF and RIPF. Moreover, IPF patients are at high risk for the development of lung carcinoma, for which radiation is often used as primary or adjuvant treatment [4,5,6,7]. Here, we examine the pathophysiological functions of mTOR in promoting IPF and RIPF and discuss potential implications of targeting mTOR in the management of fibrosis.

### 1.1. The Pathogenesis of Idiopathic Pulmonary Fibrosis (IPF)

**Figure 1:**
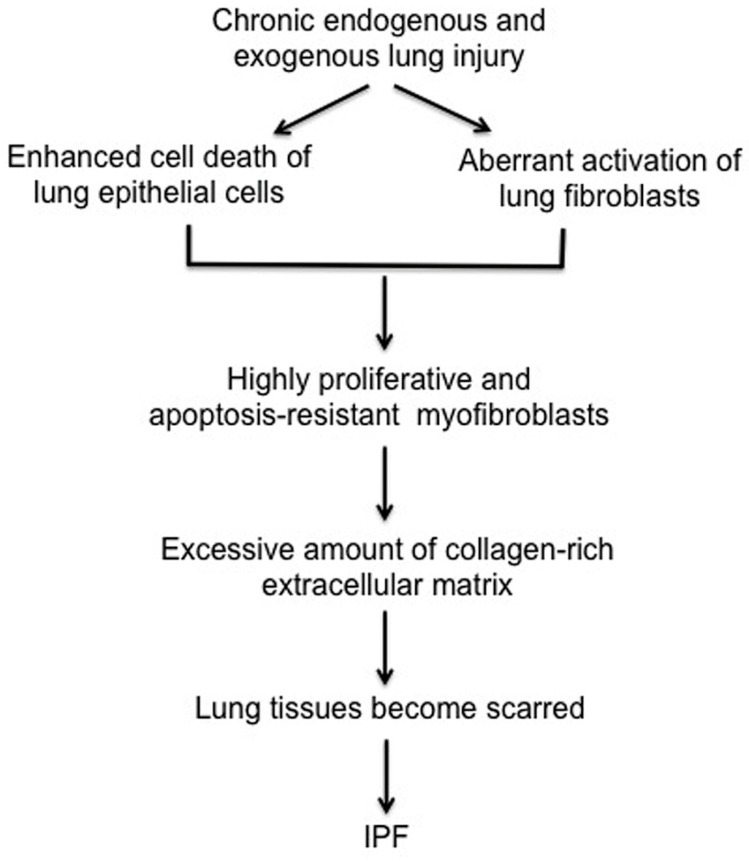
Proposed model for IPF. Chronic lung injury promotes lung epithelial cell death while (myo)fibroblasts become activated as a result of fibrosis inducing growth factors and cytokines. The failure of re-epithelization and hyper-proliferation of (myo)fibroblasts relentlessly produce collagen rich extracellular matrix, which promotes the fibrotic process, leading to the progression of lung fibrosis.

IPF is the most common type of idiopathic interstitial pneumonia and it is the prototypical chronic, progressive, and almost uniformly fatal fibroproliferative lung disease. IPF is estimated to occur with similar frequency to cancers of the stomach, brain and testicles, although true incidence is difficult to capture [8,9,10]. Patients with IPF have a characteristic “usual” interstitial pneumonia (UIP) pattern characterized by progressive scarring of lungs [11]. While pirfenidone and nintedanib were both approved by the Food and Drug Administration (FDA) to treat IPF, they help to reduce clinical exacerbations that affect pulmonary function and do not represent curative approaches [12,13]. IPF is a multifactorial disease that likely results from complex interactions between genetic and environmental factors [14,15]. Although the precise underlying mechanisms that underlie the development of IPF are not understood, a growing number of studies suggest that IPF is initiated by endogenous or exogenous lung epithelial injury (Figure 1). There are two different types of lung epithelial cells, type I and type II pneumocytes in human lung. Type I pneumocytes cover approximately 95% of lung epithelium and play a crucial role in gas exchange. Although type II pneumocytes account for small portion of the cell population, they function as progenitor cells to repair damaged lung epithelium. Lung injury can occur via several mechanisms, as it is exposed to many exogenous and endogenous insults. Under normal physiological conditions, a coordinated sequence of events triggered by growth factors and cytokines, such as TGF-β, IL-6 and IL-1β that are produced during the repair process to recruit immune cells. During this process, fibroblasts become activated to myofibroblasts and produce type I collagen-rich ECM to help repair lung epithelium. Following proper repair, fibroblasts then undergo apoptosis to maintain homeostasis. In fibroproliferative diseases, this repair process is disrupted, resulting in aberrant repair, which may contribute to chronic lung fibrosis. Studies suggest that chronic exposure to environmental factors such as cigarette smoke and environmental toxins is closely associated with the development of IPF [14,16,17]. Transforming growth factor beta (TGF-β) is a particularly well-characterized pro-fibrotic growth factor produced during lung injury. In vitro assays have clearly demonstrated that TGF-β activates fibroblasts to become myofibroblasts, leading to production of the type I collagen ECM [18,19]. The current working hypothesis is that IPF results following aberrant wound healing from an unknown and chronic lung insult that increases the production and/or activity of TGF-β and other pro-fibrotic cytokines that encourage the relentless production of type I collagen-rich ECM via (myo)fibroblast activation. This aberrant lung tissue repair process subsequently promotes the emergence of proliferative and apoptosis-resistant fibrotic fibroblasts. Eventually, collagen-rich ECM produced by these fibrotic fibroblasts distorts lung epithelium, severely disrupting normal gas exchange.

Collagen-rich ECM is a crucial underlying feature linked to lung fibrosis, therefore understanding its pathophysiological functions is key to identifying effective targets. Studies have clearly demonstrated that cell-matrix interactions profoundly affect cellular phenotype. Collagen is the main structural protein and is also the most abundant protein in mammals, making up from 25% to 35% of the whole-body protein content [20]. There are many different types of collagen, but type I collagen comprises over 80–90% of collagen in mammals [21,22]. Importantly, type I collagen is also predominantly found in human lung fibrotic tissues and likely influences signaling pathways. For example, when normal lung fibroblasts interact with type I collagen ECM, the phosphoinositide 3-kinase (PI3K)/protein kinase B (AKT) kinase pro-survival pathway is suppressed by high tumor suppressor phosphatase and tensin homolog (PTEN) activity, which inhibits fibroblast proliferation and promotes apoptosis [23,24]. In contrast, lung fibroblasts isolated from IPF patient fibrotic foci (IPF fibroblasts) have enhanced PI3K/AKT activity due to PTEN suppression, which causes IPF fibroblasts to have a highly proliferative and apoptosis-resistant phenotype on collagen matrix [24,25]. The interaction of IPF fibroblasts with collagen matrix is crucial for this increased proliferative and apoptosis-resistance to occur, as IPF fibroblasts grown without collagen matrix behave similarly to normal lung fibroblasts. Membrane-bound PTEN activity is reduced as a result of low caveolin-1 (cav-1) expression [26]. Immunohistochemical (IHC) analysis of fibroblasts within the fibroblastic foci of IPF patient specimens also revealed that PTEN, caveolin-1 (cav-1) and forkhead box O3a (FoxO3a) expression are all suppressed, while AKT activity is upregulated, further supporting the importance of the PTEN-PI3K/AKT axis in the pathogenesis of pulmonary fibrosis [26,27]. Further work needs to elucidate the progression of initial lung micro-injuries that ultimately lead to abnormally viable, matrix-producing fibroblasts.

### 1.2. The Pathogenesis of Radiation-Induced Pulmonary Injury (RIPF)

Approximately 50% of cancer patients will be prescribed radiation therapy (RT) during treatment, with tumors of the breast, prostate and lung comprising great than half of the tumor types for which radiation is indicated [9,28]. Radiation-induced lung toxicities, namely pneumonitis and pulmonary fibrosis, are relatively common following radiation treatment to thoracic structures or lower neck, either as part of the target volume or due to the proximity to the tumor target. While pneumonitis occurs early following treatment and may be reversible, pulmonary fibrosis is a delayed toxicity that can develop years following treatment [29,30]. In studies of lung cancer patients, radiation pneumonitis can occur in as many as 50% of patients, and rates of pulmonary fibrosis can be as high as 70–80% in high-dose regions of the lung [31,32,33,34,35,36]. It is currently unclear if radiation induced fibrosis results from a failure of the normal healing response in pneumonitis or represents a separate, complicating entity [37,38,39,40]. As in IPF, there is an unmet clinical need to develop effective treatment strategies that reverse RIPF. There has been an abundance of research in radiation pneumonitis, as it occurs shortly after radiation treatment and is therefore easier to study than delayed RIPF. Because the development of pneumonitis does not predict the development of RIPF, precise underlying mechanisms that promote RIPF are not understood. Clinical and dosimetric risk factors are currently considered when prescribing RT, but it is difficult to predict the true risk of radiation fibrosis, for which there exists no effective treatment [41,42,43,44,45]. Because of the risk of RIPF, lung is considered dose-limiting and may therefore restrict the overall dose of radiation that can safely be administered to a tumor, diminishing tumor control.

**Figure 2:**
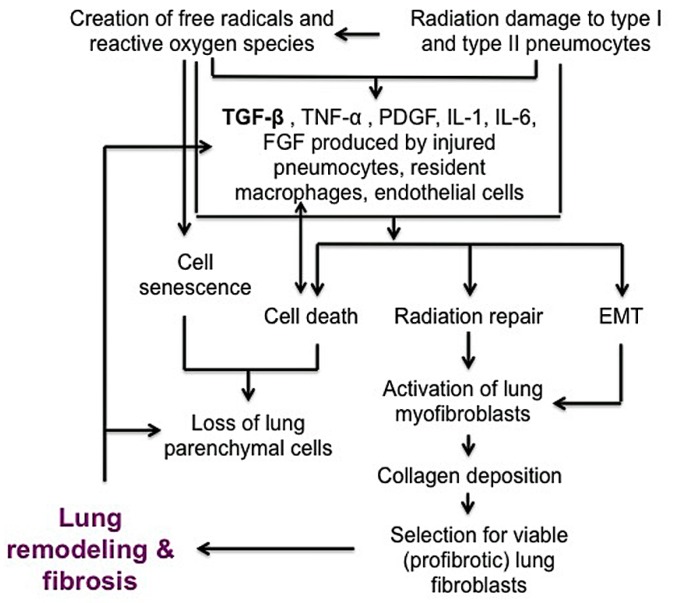
Schematic figure describing the development of radiation-induced chronic lung fibrosis. Radiation causes damage to pneumocytes and induces secondary reactive oxygen species that contribute to a pro-inflammatory environment. Acutely injured lung triggers healing mechanisms that activate (myo)fibroblasts. Excessive accumulation of collagen, which normally inhibits further fibroblast growth, selects for pro-fibrotic, viable lung fibroblasts. Fibroblast activation and selection combined with the inflammatory cytokine cascade, epithelial to mesenchymal transition (EMT), and loss of parenchymal cells leads to ongoing lung fibrosis. The bidirectional arrow indicates that cell death creates inflammatory cytokine production which further increases cell death.

Ionizing radiation induces a complex series of injuries mediated by oxidative stress, cell death or senescence and loss of normal lung barrier functions (Figure 2) [46]. Radiation injury initially injures type I pneumocytes lining the alveoli but is followed by disruption of type II pneumocytes, which secrete surfactant to prevent alveolar collapse at expiration and serve as precursors for type I pneumocytes [40,47]. Injured pneumocytes, resident macrophages and/or endothelial cells that line alveoli stimulate release of inflammatory cytokines such as TGF-β, tumor necrosis factor-alpha (TNF-α), platelet derived growth factor (PDGF), interleukin (IL)-1 and IL-6, as well as fibroblast growth factor (FGF) [38,48,49,50,51]. Similar to its pro-fibrotic role in IPF, TGF-β is a powerful central mediator of early and late lung radiation injury response, serving to activate fibroblasts and perpetuate the fibrotic cascade. Activated (myo)fibroblasts at the injured site then produce collagen-rich ECM proteins during repair of basement membranes. During normal healing, alveolar–capillary permeability is repaired and inflammation resolves. Following radiation injury to lung, inappropriate activation of myofibroblasts can result in tissue remodeling and excessive extracellular matrix deposition [48,52,53,54]. Some alveolar epithelial cells can also undergo transdifferentiation into myofibroblasts through epithelial-to-mesenchymal transition (EMT). EMT is controlled by transcriptional factors such as Snail and Twist, and the activation of these proteins represses E-cadherin and increases contractile protein α-smooth muscle actin (α-SMA) [52,53,55,56]. It is likely that this combination of continued myofibroblast activation, collagen deposition, EMT and persistent inflammatory cytokine signaling leads to fibrosis. As in IPF, collagen that is predominantly produced by fibroblasts, is the most abundant matrix within fibrotic lesions. It is also well recognized that collagen metabolism is frequently deregulated in neoplastic disease, contributing to an altered ECM [57,58]. Like IPF fibroblasts, cancer-associated fibroblasts present within tumor microenvironment also play a significant role in disease progression and are a clear target for treatment [59,60]. While the focus of this review is on the mechanisms of pulmonary fibrosis, there are several excellent reviews that examine the role of cancer-associated fibroblasts in modulating the tumor microenvironment [61,62,63,64].

Cellular consequences of radiation are variable and depend on multiple factors. Simplistically, radiation induces both single strand and double-strand DNA damage, which triggers the DNA damage response pathway. Following activation of the damage response, cell cycle checkpoints are triggered to halt cell cycle progression to allow repair or initiation of cell death or senescence [65,66]. Ataxia telangiectasia mutated (ATM) protein, the primary regulator of the DNA damage response following ionizing radiation, phosphorylates target proteins involved in the cell cycle, DNA repair, apoptosis and senescence that ultimately signal the cell to undergo DNA repair, cell death or senescence [67].

### 1.3. The Role of mTOR in Pulmonary Fibrosis

In normal cells, the PI3K/AKT kinase pathway is a central signaling regulator of cell metabolism, proliferation, differentiation, and survival [68]. The pathway is often deregulated in a vast array of diseases, including solid tumors, immune-mediated disease and idiopathic pulmonary fibrosis, and therefore represents an attractive therapeutic target [27,69,70]. The pathway is activated by cell surface receptors such as tyrosine kinase receptors that active the p110 subunit of PI3K. This catalyzes the conversion of phosphatidylinositol 4,5-bisphosphate (PIP_2_) to phosphatidylinositol 3,4,5-triphosphate (PIP_3_) to activate AKT. AKT signals to several downstream effectors including mammalian target of rapamycin (mTOR), forkhead transcription factors (Fox proteins), and Bcl-2 anti-apoptotic protein. mTOR is a serine/threonine kinase in the PI3K family that is an important regulator of protein and lipid biosynthesis, cell cycle progression, proliferation, survival, and senescence [54,71,72,73]. While the PI3K/AKT/mTOR pathway is well described, it is increasingly realized that protein kinase interactions are complex with many feedback loops that each mediate separate cellular processes [69,71,74]. The importance of mTOR within this signaling network was first realized with the knowledge that rapamycin possessed marked antiproliferative properties through its ability to inhibit signaling pathways required for cell growth and proliferation [75,76]. Recognizing its key role in growth and proliferation, researchers have since found that mTOR is involved in a vast array of other cellular processes, including (but not limited to) metabolism, inflammation, apoptosis and senescence. The central role of mTOR in regulating many diseases, including fibrosis and cancer, therefore establish it as a highly valuable target for manipulation [77,78].

## 2. mTOR

**Figure 3:**
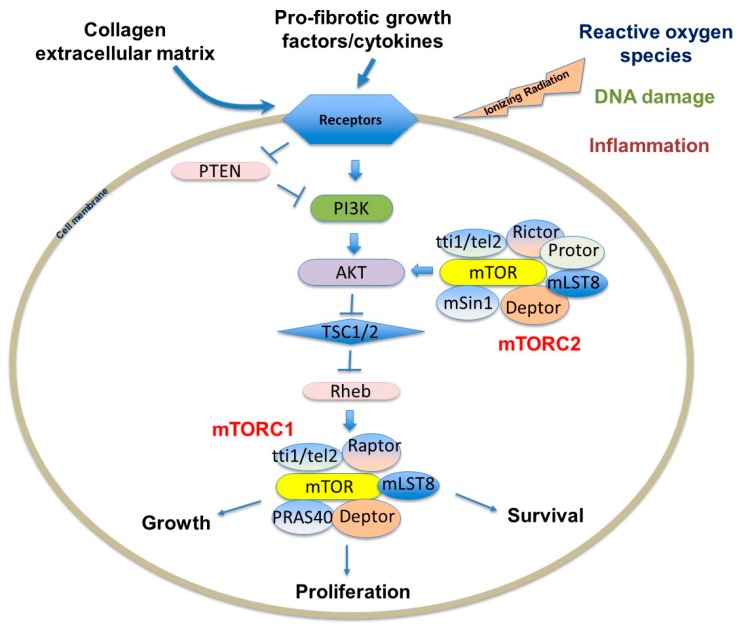
mTORC1 and mTORC2 complexes and environmental signals to regulate the PI3K/AKT/mTOR pathway to influence growth, proliferation and survival. T indicates the inhibition of the target molecule.

mTOR interacts with several proteins to form two distinct complexes: mTORC1 and mTORC2 (Figure 3). Each complex has a different set of upstream regulators and downstream targets and respond to rapamycin uniquely [77,78]. While mTORC1 controls cell growth and metabolism and is highly sensitive to rapamycin, mTORC2 regulates cell proliferation and survival and is relatively insensitive to rapamycin [77,79]. mTORC1 is impacted by a variety of pathways, including those involving growth factors, cell stress, hypoxia and DNA damage [77,79,80]. Growth factors and mitogen-dependent signaling pathways all inhibit Tuberous Sclerosis Complex (TSC), which is a major negative regulator of mTORC1 [78,81]. While the mTORC2 pathway is not as well understood as mTORC1, it appears to be predominantly regulated by growth factors through PI3K [77,82]. AKT activation suppresses TSC1 and TSC2, which indirectly activates mTOR kinase activity through the GTP binding protein Ras homolog enriched in brain (Rheb). Studies validating this pathway have shown that TSC-deficient cells have constitutive Rheb-GTP, leading to high mTORC1 activity [81,83]. As the mTOR axis is regulated by upstream PI3K/AKT signals, which are aberrantly active in several solid tumors, enhanced mTOR activity is also found in those patients, creating a promising cancer therapeutic target [69,71,84,85,86].

### 2.1. The Structure and Function of mTORC1

The mTOR complexes have overlapping and unique subunits that contribute to signaling activity. mTORC1 contains mTOR, regulatory-associated protein of mTOR (Raptor), mammalian lethal with SEC13 protein 8 (mLST8)/G-protein β-subunit-like protein (GβL), PRAS40, DEPTOR and scaffold protein TTI1/TEL2 complex [87]. PI3K-dependent pathway activation leads to increased collagen I expression via the mTORC1-dependent 4E-BP1/eukaryotic translation initiation factor 4E (EIF4E) signaling [87]. Raptor is known to facilitate substrate recruitment to mTORC1 and is required for the correct subcellular localization of mTORC1 [88,89]. MLST8 associates with the catalytic domain of mTORC1 and thought to stabilize the kinase activation loop [90]. There are two inhibitory subunits, PRAS40 (proline-rich AKT substrate of 40 kDa) and DEPTOR (DEP domain containing mTOR interacting protein) [91,92,93]. Once activated, mTORC1 phosphorylates several effectors, the most common including S6 kinase 1 (S6K1) and 4E-BP1 to promote protein translation [74,78,87]. mTORC1 primarily regulates cell growth and autophagy in stressful environments, which alters fibroblast proliferation and viability. Since type I collagen-cell interactions play a crucial role in the progression of lung fibrosis, abnormal mTORC1 regulation is therefore important in many chronic fibroproliferative diseases.

### 2.2. The Structure and Function of mTORC2

The mTORC2 complex is not as well described as the mTORC1 complex, but research continues to unravel its role in proliferation and survival. mTORC2 is composed of mTOR and the rapamycin-insensitive companion of mTOR (RICTOR), mLST8/GβL, mammalian stress-activated protein kinase interacting protein 1 (mSIN1), Protor 1/2, DEPTOR, TTI1 and TEL2 [78,87]. Signaling through mTORC2 is insensitive to nutrients but does respond to growth factors such as insulin through a poorly defined, PI3K-dependent mechanism [94]. Interestingly, mTORC2 activates AKT, which subsequently activates the mTORC1-dependent pathway. Recent experiments also suggest that mTORC2 may be associated with fibroblast pathogenesis through a TGF-β-dependent pathway [74,95]. TGF-β was shown in one study to induce Rictor in IPF lung fibroblasts, subsequently activating mTORC2 signaling and AKT [95]. It is feasible that the AKT activity in pathological fibroblasts is abnormally activated through mTORC2 signaling, contributing to the highly viable, apoptosis-resistant fibroblast phenotype identified in IPF fibrotic foci. Further highlighting the complexity of mTORC interactions, while the most important role of mTORC2 is the activation of AKT, which can activate mTORC1, mTORC2 signaling is also regulated by mTORC1; mTORC1 activation is known to indirectly suppress mTORC2 through growth factor receptor-bound protein 10 (Grb10) and S6K1 signaling [78,96,97,98,99].

## 3. mTOR-Dependent Molecular Mechanisms that Promote Pulmonary Fibrosis

### 3.1. mTOR Regulates Cell Growth, Proliferation, and Viability

Although the definition for the cell growth and proliferation has been used interchangeably, it is important to highlight the difference between the two processes. Cell growth indicates an increase in mass while cell proliferation implies increase in cell number. There is correlating growth and proliferation due to unidirectional coupling such that growth must happen in order for cell-cycle progression to occur, but the cell cycle itself does not promote growth [100]. mTOR signaling is associated with both cell growth and proliferation. As previously discussed, mTOR binds to S6K1 and 4E-BP1, recruits raptor, and activates mTOR-dependent signaling [88]. In one study, increased mTOR kinase activity increases EIF4e function in colon cancer cells to promote proliferation [101]. In a separate study of human melanoma, inducible nitric oxide synthase overexpression increased mTOR activity, resulting in increased cell proliferation [102]. Several studies have shown that PI3K/AKT signaling is associated with cell proliferation and not cell growth [103,104,105,106]. The p110α catalytic subunit of PI3K is frequently mutated and becomes activated in many cancer cells [107,108,109]. Both proliferation and cell mass were studied in dermal fibroblasts with endogenous p110α subunit mutations, and increased proliferation but not hypertrophy was noted [110]. In contrast, when mTORC2 was inhibited in mesangial cells, mTORC1 was activated and mesangial cell hypertrophy increased [111]. Endoplasmic reticulum (ER) stress is also implicated in the development of lung fibrosis via the activation of PI3K/AKT/mTOR-dependent signaling [112]. Treatment with ER inhibitors or PI3K inhibitors caused a reduction in fibroblast proliferation and improved pulmonary function in one study [112]. Collectively, data suggests that mTOR-dependent cell proliferation and growth may be cell type-dependent. Like cancer cells, mTOR seems to play an important role in increasing proliferation in various types of fibroblasts. In one study, investigators showed that mTOR promoted keloid fibroblast (KF) proliferation [113]. Moreover, the dual mTORC1 and mTORC2 inhibitor Palomid 529 (P529) exerted anti-keloid disease (KD) effects in a novel KD organ culture assay and in KF cells [113]. As PI3K/AKT/mTOR/S6K1 signaling is required for ATP-induced proliferation in adventitial fibroblasts, this provides evidence supporting that mTOR is central to modulating fibroblast proliferation.

**Figure 4:**
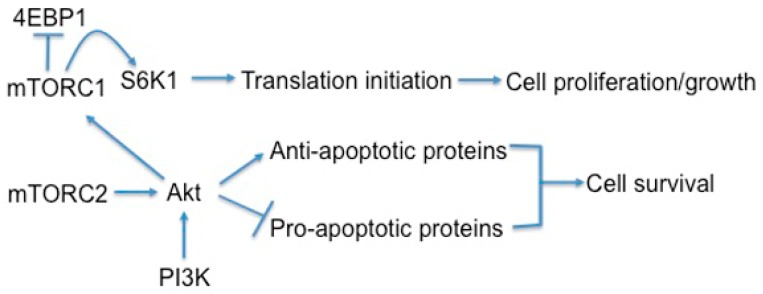
mTOR regulates cell growth, proliferation, and survival. The arrows indicates positive regulation while the symbol T indicates the inhibition of the target molecule(s).

Cell survival is also dependent on mTOR signaling, particularly through mTORC2 and its phosphorylation and activation of AKT, a key effector of PI3K survival signaling (Figure 4) [80,114]. However once cannot discount that mTORC1 signaling also plays a role in modulating survival. mTORC1 is a major player in metabolism, and inhibition of mTORC1 can lead to increased autophagy and macropinocytosis, therefore permitting survival in poor nutrient conditions [78,115]. In support of this notion, when non-IPF fibroblasts were treated with autophagy inhibitors in the absence of serum, they become sensitized to collagen matrix driven cell death [116]. In contrast, IPF fibroblasts maintained their viable phenotype under the same conditions. This study showed that the aberrant PTEN/AKT/mTOR axis desensitizes IPF fibroblasts from collagen matrix-driven stress by suppressing autophagy, which produces a viable IPF fibroblast phenotype on collagen [116]. Since mTORC1 activation is also known to indirectly inhibit mTORC2, the inhibition of mTORC1 may remove the negative feedback loop on mTORC2/AKT, thus paradoxically promoting cell survival.

### 3.2. DNA Damage, Radiosensitivity, and DNA Damage Response

**Figure 5:**
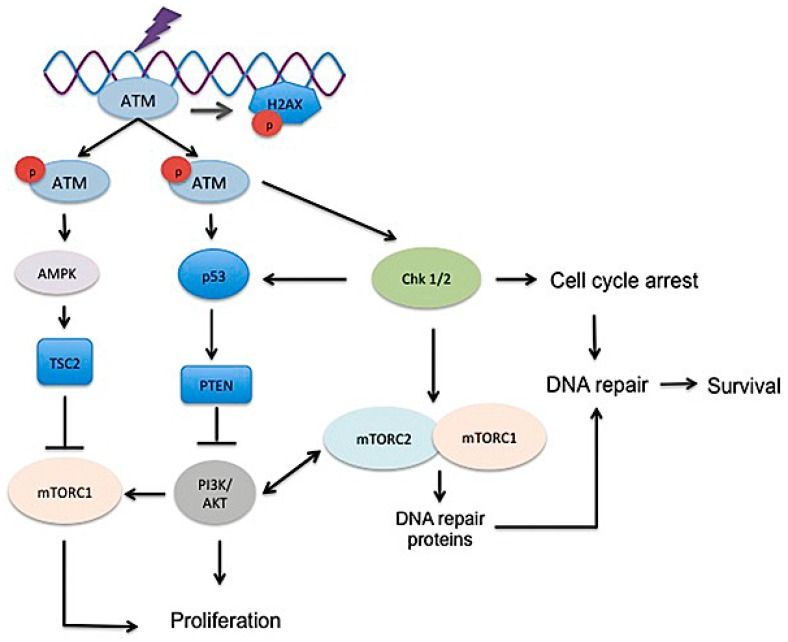
Proposed mechanism by which mTOR may contribute to radiosensitivity and DNA damage repair and thereby potential means in which inhibition of mTORC1 or mTORC2 may alter cell cycle arrest, DNA repair and cell survival following radiation. Pathologic pro-fibrotic lung fibroblasts may depend on both mTORC1 and mTORC2 for efficient cell cycle arrest and repair of DNA damage following radiation damage. In non-radiation induced lung damage, DNA damage may result from various chemical or other microinjuries that create a similar population of fibroblasts that depend on mTOR complexes for survival and proliferation. The bidirectional arrow indicates that AKT activates mTORC2 while mTORC2 can also positively impact PI3K/AKT signaling. T indicates the inhibition of the target molecule. The purple “bolt” indicates ionizing radiation.

It is easiest to discuss the role of mTOR in the context of radiation-induced DNA injury as the main mechanism of radiation cell killing is via the creation of DNA double-strand breaks (DSB). Mechanisms of DNA repair apply to more than simply radiation damage and may help to explain the highly viable lung fibroblast phenotype identified in fibrotic lesions. The role of mTOR in determining radiation response is not fully understood and is likely dependent on many factors such as cell type, microenvironment, and competing extracellular or intracellular signals (Figure 5). Rapamycin and other mTOR inhibitors have antitumor activity and act as radiosensitizers in many solid tumors [86,117,118,119,120,121,122]; however, they are also radioprotectors in several normal cell types in vitro [72,123,124]. The precise role played by each mTOR complex is not clear. Intracellular and/or extracellular stressors such as hypoxia or DNA damage generally downregulate mTORC1, limiting cell growth and metabolic functions [78]. However, the activation of mTOR in periods of stress—such as after radiation exposure—can encourage accelerated cell death rather than cell cycle arrest, as essential nutrients may not be available to the cell [123,125,126]. Cancer cells are often capable of surviving in abnormal and harsh conditions such as hypoxic and low nutrient conditions and therefore may have altered mTOR regulators or a shift in dependence on the PI3K/AKT/mTOR pathway. DNA damage in normal cells triggers p53 activation and the induction of p53 target genes such as the *AMPK* subunit, *PTEN*, and *TSC2* ultimately increases TSC activity to subsequently inhibit mTORC1 to halt cell growth [79,127,128]. In a study investigating murine pluripotent stem cells, knockdown of either mTORC1 or mTORC2 reduced radiation-induced apoptosis, suggesting that both complexes play a role in radiation response [123]. Interestingly, in studies of lung cancer, mTORC1 inhibition by rapamycin caused G1 arrest even in p53-deficient cells and increased radiosensitivity in all cell lines [121]. The ability of rapamycin to act as both radiosensitizer and radioprotector may be a result of its lack of impact on mTORC2. For example, in cells with altered PI3K signaling, such as cancer cells or pathologic IPF fibroblasts, mTORC1 inhibition may allow uninhibited mTORC2 activity, further suppressing mTORC1 but increasing phosphorylation of AKT and its downstream transcription factors, thus promoting cell survival and proliferation [78,95]. mTORC2 is sensitive to growth factors rather than nutrients, therefore the advent of novel mTORC1/mTORC2 inhibitors may provide better modulation of survival following radiation or chemical-induced DNA damage in pathologic cells with deregulated PI3K/AKT/mTOR signaling [87,95,129,130,131]. Importantly, dual mTORC1/mTORC2 inhibitors decreased radiation-induced apoptosis in murine pluripotent cells, suggesting that even though multiple targets in the PI3K pathway are hit, normal cells may not sustain enhanced injury [123]. Other studies have also shown that multiple PI3K inhibitors, which also inhibit mTOR, mitigate radiation damage to normal cells in vitro and in vivo, highlighting the pivotal role this pathway has in determining radiation response [85,132].

Tumor cells generally possess impaired DNA repair capabilities than normal cells, thus making them more susceptible to radiation-induced DNA damage [133,134]. This supports the observation that mTOR signaling and inhibition induces differential responses on tumor cell repair compared to normal cell repair. In one study evaluating the effect of radiation on hair follicle transit amplifying cells, radiation induced mTORC1 activation until full regeneration of the hair follicle was complete [135]. Moreover, inhibiting mTORC1 by rapamycin increased radiation-induced cell apoptosis and reduced cell proliferation, leading to hair loss in the irradiated mice. Results suggest that mTORC1 is necessary for efficient repair of injured hair follicles to occur following radiation [135]. Pathologic fibrotic lung fibroblasts obtained from patients with IPF resist stress-induced apoptosis through abnormally high PI3K/AKT/mTOR activation that results from PTEN suppression [24,27,136]. High mTORC1 and mTORC2 activity may therefore translate to improved DNA repair, permitting survival and proliferation of fibroblasts that favor and encourage fibrosis. As these pathologic fibroblasts have altered cell signaling, mTOR inhibitors may increase fibroblast cytotoxicity following radiation, thus mitigating fibrosis. Indeed, in a murine model of radiation-induced pulmonary fibrosis, rapamycin treatment following coarse-fractionated thoracic radiation reduced lung collagen accumulation compared to irradiated control mice that did not receive rapamycin [72].

Although there is little evidence to suggest that mTOR directly affects DNA repair proteins, mTOR may indirectly alter DNA repair as it regulates several genes involved in the DNA damage response and cell cycle machinery [130,137,138,139]. Studies in cancer cells have shown that radiation induces mTOR signaling and that inhibition of mTOR alters cell cycle progression, apoptosis and repair to increase radiosensitivity [140,141]. The predominant lethal event following radiation in non-hematopoietic cells is the production of DNA double-strand breaks, which typically induce mitotic catastrophe and apoptosis [142,143,144,145,146]. Under normal conditions, double-strand breaks trigger a cascade of events involving ATM and phosphorylated (γ-) H2AX that encourage repair at the site of DNA damage [147]. Several studies have provided evidence that mTORC1 and mTORC2 both function to protect against DNA damage in tumor cells [139,141,148]. Other studies have demonstrated that the mTOR inhibitor everolimus may sensitize tumor cells to apoptosis via p21 inhibition and inhibit cell proliferation following DNA damage [138,149,150]. The induction of DNA damage leads to mTORC1 inhibition through p53 signaling; however, there are also studies that show mTORC1 positively regulates p53 to alter cell cycle progression [128,151]. In a recent study investigating radiation damage in breast cancer cells, both mTORC1 and mTORC2 were required to enable DNA damage repair and cell survival [141]. Both mTOR complexes coordinated transcription and translation of genes involved in cell cycle, DNA replication, recombination and repair [141]. Moreover, the study demonstrated that dual mTOR inhibition but not mTORC1 inhibition alone delayed DNA damage repair in irradiated cells, as demonstrated by increased γ-H2AX expression and ATM, DNA-PKcs, and CHK2 phosphorylation [141]. Combined Raptor and Rictor silencing with radiation also induced sustained DNA double-strand breaks and activation of DNA damage repair signaling, while selective silencing of Raptor or Rictor did not [141]. In a separate study investigating DNA damage in breast carcinoma cells, dual mTOR inhibition prevented Chk1 activation and DNA damage-induced S and G2/M cell cycle arrest [139]. Finally, two other studies have identified that mTORC1 upregulates *FANCD2* gene expression in cancer cells, which is important for DNA double-strand break repair [152,153]. Results such as these directly inform studies looking to inhibit mTOR signaling. While it is unclear if pathologic fibroblasts behave similarly to cancer cells, it warrants an investigation to determine the role of mTORC1 and mTORC2 on cell cycle arrest and DNA repair across different cell types.

Homologous recombination repair following DSB is a primary, high-fidelity mechanism of radiation repair in human cells. The recruitment of the repair proteins RAD51 and breast cancer-associated gene 2 (BRCA2) to the damaged DNA sites is crucial to this repair pathway [154,155]. FoxM1, a member of the forkhead family of transcription factors, is known to upregulate both RAD51 and BRCA2, thereby protecting cells from radiation-induced DNA damage [156,157]. Irradiated murine lung and human IPF fibrotic lesions both demonstrate increased FoxM1; the conditional deletion of FoxM1 prevented lung fibrosis in a murine model of radiation fibrosis [158]. There is a negative feedback loop between FoxM1 and FoxO3a, as prior studies have shown that FoxM1 activation occurs following FoxO3a suppression [156,159]. It has been well documented that FoxO3a is aberrantly suppressed in IPF fibroblasts and IPF patient lung tissues [24,27,160,161]. Since AKT is a primary kinase that regulates FoxO3a and mTORC1 activity, it is therefore likely that FoxO3a activity is inversely correlated with mTOR activity in corresponding tissues [24,27,116,160].

### 3.3. Inflammation

**Figure 6:**
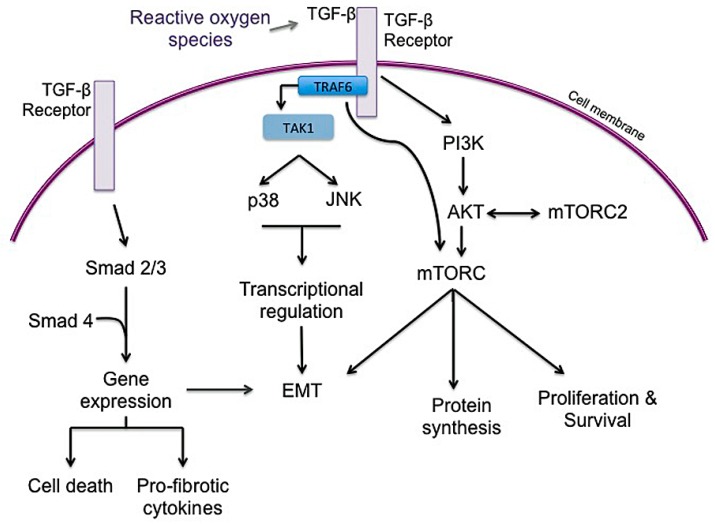
Schematic describing potential interactions of mTOR and TGF-β, a major driver of lung fibrosis. Both mTORC1 and mTORC2 likely plays a role in pulmonary fibrosis although the exact contribution from each complex is not clear. Both the canonical (Smad) and non-canonical (non-Smad) pathways may drive ongoing lung fibrosis, including altering metabolism, epithelial to mesenchymal transition (EMT), inflammation, and proliferation. It is possible that different mechanisms are predominate within different cell types to promote fibrosis; for example, Smad-dependent TGF-β signaling may trigger apoptosis in lung epithelial cells yet pro-fibrotic signals predominate in fibroblasts.

The underlying mechanism of RIPF is not fully understood, but radiation is thought to cause oxidative stress and free radical production that leads to DNA damage and an inflammatory response in tissue. While IPF may no longer be considered a chronic inflammatory disorder that gradually progresses to fibrosis, it is considered a disease resulting from micro-injuries to pneumocytes, resulting in aberrant healing and the induction of collagen-producing myofibroblasts [10]. Radiation injury induces significant changes in cytokine, chemokine, and prostaglandins that promote inflammation in both normal tissue and tumor tissue. Early response cytokines such as TNF- α and IL-1α, IL-1β, and IL-6 are strong pro-inflammatory cytokines that trigger inflammatory cells to infiltrate irradiated tissue [38,162,163]. TGF-β, IL-1β, and IL-6 are recognized as major drivers of radiation-induced lung fibrosis and play significant roles in IPF progression and exacerbations (Figure 6) [3,49,51,72,164,165,166]. There is plenty of evidence to support that TGF-β is a powerful central mediator of radiation injury [37,39,46,49,54]. Radiation causes a dose-dependent increase in TGF-β activity in tissue within minutes to hours after radiation [51]. TGF-β may normalize after radiation but increase again with chronic radiation injury to normal lung [51]. TGF-β is typically secreted as a latent cytokine that is activated after specific stimuli such as exposure to radiation, oxidative stress or proteases [46,167,168,169]. Similar to radiation, TGF-β is considered a primary player in IPF progression, as IPF may represent a chronic disease state that occurs after an initial, often unknown, lung injury [3,10,165]. Active TGF-β ligands are then capable of binding to several TGF-β receptors to exert pleiotropic biological effects. TGF-β1 receptor signaling through Smad proteins is the most well-described and results in the regulation of many genes involved in epithelial-mesenchymal transition (EMT), immune suppression, cell proliferation and inflammation [39,46,51,54]. Within normal tissue such as lung, Smad signaling can stimulate fibroblast proliferation and collagen deposition, creating a hypoxic environment, which may further increase mTOR signaling to encourage cell survival and fibrosis [46]. Smad-independent TGF-β signaling pathways also operate by several other mediators involved in inflammation and proliferation, including TGF-β-associated kinase 1 (TAK1), extracellular signal-regulated kinase (ERK), mitogen activated protein kinase (MAPK), AKT, and JNK, [46,170,171,172,173,174]. TGF-β-mediated AKT signaling, downstream of PI3K may further activate mTOR signaling. TAK1 is a MAPK kinase kinase member that is important in sensing environmental changes, and it triggers downstream kinases to alter cell growth and metabolism, inflammatory responses, EMT and tumor invasion [172]. Importantly, TAK1 controls downstream p38 MAPK signaling, which promotes cardiac hypertrophy and atrial fibrosis [174,175]. Recent studies have highlighted the role of tumor necrosis factor receptor-associated factor (TRAF) family in pathological cardiac remodeling [173,174,175,176,177,178]. TRAF6, in particular, is a critical activator of TAK1 and has been highlighted as a potential target in cardiac hypertrophy and fibrosis [173,174,175,179,180]. Notably, the production of reactive oxygen species (ROS), such as in atherosclerosis, activates TRAF6 to induce cardiac remodeling [174]. Recognizing the importance of ROS in ongoing fibrosis (Figure 2), TRAF6 may represent a biomarker for severity of disease as well as a therapeutic target in pulmonary fibrosis.

Indeed, there is some suggestion that patient plasma TGF-β levels before or during radiation therapy may help predict radiation toxicity with higher levels being associated with higher risk [30,181,182]. Many cell types are involved in the perpetuation of TGF-β-signaling, including macrophages, activated fibroblasts, and fibrosis-associated fibroblasts [46]. It is clear that permitting increased survival of fibrosis-associated secretory fibroblasts through mTORC2 signaling further encourages fibrosis. TNF-α is important in mediating early tissue responses to radiation, as it is a pro-inflammatory cytokine that is both rapidly expressed in irradiated tissue and linked to acute toxicities [183,184,185]. In a murine model of radiation-induced lung injury, TNF-α knockout mice displayed a lower, asymptomatic degree of radiation pneumonitis compared to wild-type mice [184,185,186]. There may be a complex relationship between TNF-α and TGF-β; both cytokines are robustly increased following ionizing radiation [169,187]. However, there is some evidence that high dose-rate and high dose targeted radiation aimed at disrupting vasculature may result in lower TGF-β activity despite increased TNF-α activity [46,188,189]. Disrupting this balance may be important in ensuring adequate tumor response in cancer patients through TNF-α while limiting fibrosis via lowered TGF-β activity. When considering the targeted inhibition of TGF-β, it is also important to note that it is also a potent anti-inflammatory mediator with decreased TGF-β or TGF-β1-signaling linked to other inflammatory and autoimmune syndromes [46].

Activation of mTORC1 regulates inflammatory responses in inflammatory cells such as monocytes and macrophages [190]. In one study that investigated granulomatous disease, mTORC1 inhibited apoptosis and encouraged macrophage proliferation to promote granuloma formation [191]. While macrophages play an important role in wound healing and repair, they may contribute to lung fibrosis as part of a robust dysregulated repair process following radiation lung injury [54,192]. While they may be directly involved in radiation damage repair, they also indirectly promote fibrosis as a prominent producer of TGF-β [49,51]. If IPF results from chronic micro-injuries, it is likely that macrophages also play a role in promoting ongoing pro-fibrotic signals as part of disrupted healing.

### 3.4. Epithelial to Mesenchymal Transition (EMT)

**Figure 7:**
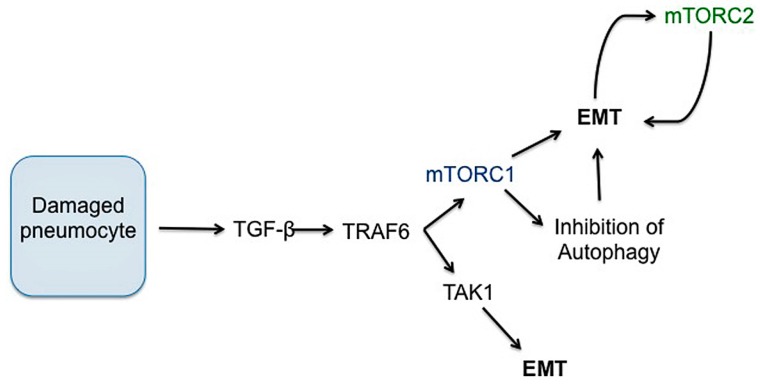
Potential roles of mTORC1 and mTORC2 in the development of EMT. TGF-β is important for the transdifferentiation of epithelial cells into fibroblast or myofibroblast-like cells. The exact roles of each mTOR complex are not clear in pulmonary fibrosis, but it is possible that the non-canonical TGF-β pathway contributes to mTORC1 activation, which induces EMT in lung epithelial cells near the site of injury. mTORC2 activity is induced in some cells undergoing EMT and may help ensure progression through the process. Rapamycin (mTORC1 inhibition) is capable of reversing EMT in some cells, suggesting transdifferentiation is disrupted.

There is likely a complex relationship between lung injury, chronic inflammatory signaling and EMT. EMT has been shown to contribute to collagen-producing fibroblasts in experimental models of pulmonary fibrosis [158,193,194]. TGF-β1 is crucial in the transdifferentiation of epithelial cells into cells with fibroblast or myofibroblast properties, a process that contributes to fibroproliferative disease (Figure 7) [158,195,196]. Several studies have highlighted that injured lung epithelial cells are an important source of TGF-β, which induces the expression of αvβ6 integrin to further increase activated TGF-β locally [196,197]. This upregulated and sustained local TGF-β production in injured lung may drive differentiation of neighboring cells into collagen-producing pathologic fibroblasts, further contributing to lung fibrosis. Notably, TRAF6 has been shown to be essential in the non-canonical TGF-β signaling pathway and in one study, was required for TGF-β-induced EMT [180]. TRAF6 also activates and helps regulate mTORC1 activation, modulating autophagy [198].

EMT is important in cancer progression and metastasis and several studies have shown that EMT is disrupted following inhibition of PI3K/AKT/mTOR, although the exact mechanism by which mTOR signaling directly modulates EMT is not clear across all pathologic processes [199,200,201,202]. mTORC2 activity is induced in cells undergoing EMT and it appears to control the progression of epithelial cells through the process [201]. However, as rapamcyin is capable of reversing EMT in some cells, there is clearly a role for mTORC1 as well, possibly through S6K signaling [203]. mTORC1 inhibition may alter metabolic processes sufficiently in neoplastic epithelial cells to inhibit the ability of cells to transdifferentiate [203]. In contrast, a separate study that investigated EMT in mammary epithelial cells determined that mTORC1 may be important in maintaining epithelial phenotype while mTORC1 inhibition increased transcription factors that trigger EMT [204]. Importantly, in this study, mTORC1 blockade induced EMT through microRNA signaling, independent of TGF-β signaling [204]. While much focus on EMT revolves around cancer research, further research needs to elucidate primary pathways that regulate EMT in fibrosis to optimize the potential for therapeutic intervention.

### 3.5. Autophagy

Recent studies have highlighted the pathological functions of mTOR-dependent autophagy in the development of pulmonary fibrosis. Although there are several types of autophagy, our review will focus on macroautophagy. This autophagic pathway consists of several distinct steps, resulting in the sequestration of cellular cargo such as damaged organelles, protein aggregates, or pathogens by the double-membrane autophagosomes [205,206]. Although the beneficial roles of autophagy are associated with the homeostatic turnover of damaged cellular organelles and proteins, deregulated autophagy is also associated with several human diseases including cancer, neurodegenerative disorders, and inflammatory bowel diseases [206,207,208]. mTOR activity may be deregulated in IPF fibroblasts, leading to the proliferative and apoptosis-resistant fibroblast phenotype through altered autophagic activity. Indeed, mTOR activity is increased in IPF fibroblasts cultured on type I collagen as a result of increased AKT activation [160]. In contrast, non-IPF fibroblasts showed low mTOR activity when cultured on collagen due to AKT suppression. It is possible that lung fibroblasts derived from IPF patients have altered responses to unfavorable conditions, and therefore maintain a stress-resistant phenotype through mTOR-dependent abnormal autophagic activity. Although PI3K/AKT plays a critical role in autophagy regulation, autophagy is also regulated by the activation of the adenosine monophosphate (AMP)-activated protein kinase (AMPK). AMPK becomes activated when AMP levels are increased under stressful conditions like serum starvation. In response to elevated intracellular AMP levels, AMPK inhibits mTORC1-dependent ULK (UNC-51 like kinase) activity by phosphorylating S317 and S777, leading to activation of autophagy [209,210,211,212]. These studies indicate that cells utilize multiple mechanisms to efficiently regulate autophagy in response to various stimuli.

**Figure 8:**
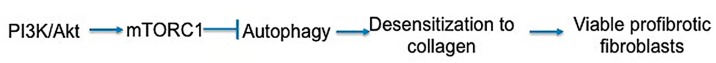
PI3K/AKT dependent autophagy regulation in IPF fibroblasts. The arrows indicates positive regulation while the symbol T indicates the inhibition of autophagy.

IPF is an age-associated disease. There is a progressive reduction of biological functions and resistance to multiple stressors during the aging process. Moreover, while aging is associated with IPF, aging-dependent autophagy alteration is also likely linked to IPF [102]. AKT activity is abnormally high in IPF fibroblasts derived from elderly patients, supporting that mTOR-dependent autophagy is also likely altered (Figure 8). Of note, autophagy was not induced in IPF biospecimens and primary IPF fibroblasts in two separate studies [116,161,213]. When IPF fibroblasts are cultured on collagen, autophagy is low as a result of activation of mTOR, while normal lung fibroblast attachment to collagen increases autophagy due to suppression of mTOR activity [116,161]. IPF fibroblasts demonstrate relatively reduced LC3B-II expression, a marker of autophagy, in response to stressful conditions when compared to age-matched control fibroblasts [116,161]. This alteration affects fibrotic IPF fibroblast proliferation and viability, and mTOR inhibition greatly sensitized IPF fibroblasts that autophagy was re-activated to collagen-induced cell death. It is thought that normal lung fibroblasts view type I collagen as a stressful, apoptosis-triggering environment while IPF fibroblasts are desensitized to collagen. Thus, these findings indicate that the desensitization of IPF fibroblasts to an unfavorable environment is an important concept that may explain IPF pathogenesis. Inappropriately high mTOR activity may alter autophagic activity to help IPF fibroblasts or other fibrotic fibroblasts maintain an apoptosis-resistant phenotype despite a stressful microenvironment.

Unlike autophagic activity in IPF, it is not yet clear whether a consistent deregulation of autophagy strictly correlates with radiosensitization. Typically, ATM signaling following radiation-induced DNA double-strand breaks reduces autophagy through decreased mTOR phosphorylation [214,215]. In some studies, increased autophagy is associated with radioresistance while inhibition of autophagy through mTOR inhibitors can increase radiosensitization [216,217,218]. It is important to recognize that most autophagy is studied in cancer cells or in the tumor microenvironment. It is feasible that resident lung fibroblasts present in an irradiated lung field at the time of treatment subsequently undergo sufficient changes by autophagy modulation that drive a phenotype similar to IPF fibroblasts. Additional studies will clarify the pathophysiological roles of autophagy in RIPF.

### 3.6. Metabolism

**Figure 9:**
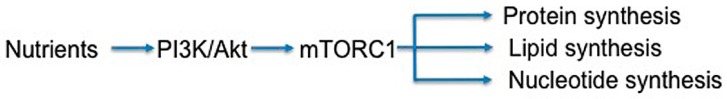
The mTOR-dependent metabolic pathway.

Tissue homeostasis is dependent on metabolism, and in diseased tissue, altered metabolism may greatly alter cell signaling. Generally, tumor cells are metabolically abnormal and can alter sources for energy as needed to ensure survival in conditions such as hypoxia or oxidative stress [219]. While the role of metabolism in modulating tumor response has been well described, non-neoplastic diseased tissue may also have metabolic dysregulation similar to those seen in cancer. Increased hypoxia-inducible factor 1α, vascular endothelial growth factor, and TGF-β signaling and hypoxia have been identified in irradiated normal tissue, which likely alter the microenvironment [54,220,221,222]. This altered microenvironment may encourage irradiated fibroblasts to utilize compensatory metabolic pathways to overcome injury. In studies utilizing hyperpolarized ^13^C-pyruvate magnetic resonance spectroscopy to examine the conversion of pyruvate to lactate, irradiated lung demonstrated higher lactate signal compared to unirradiated normal tissue, which correlated to macrophage inflammation and early radiation-induced injury [223,224]. Recognizing the role that mTORC1 plays in modulating cellular response to nutrient availability, targeting the mTOR pathway may disrupt the response to injury (Figure 9). However it will be difficult to tease out the exact role of metabolic inhibition with mTOR inhibitors given their impact on downstream effectors.

### 3.7. Senescence

**Figure 10:**
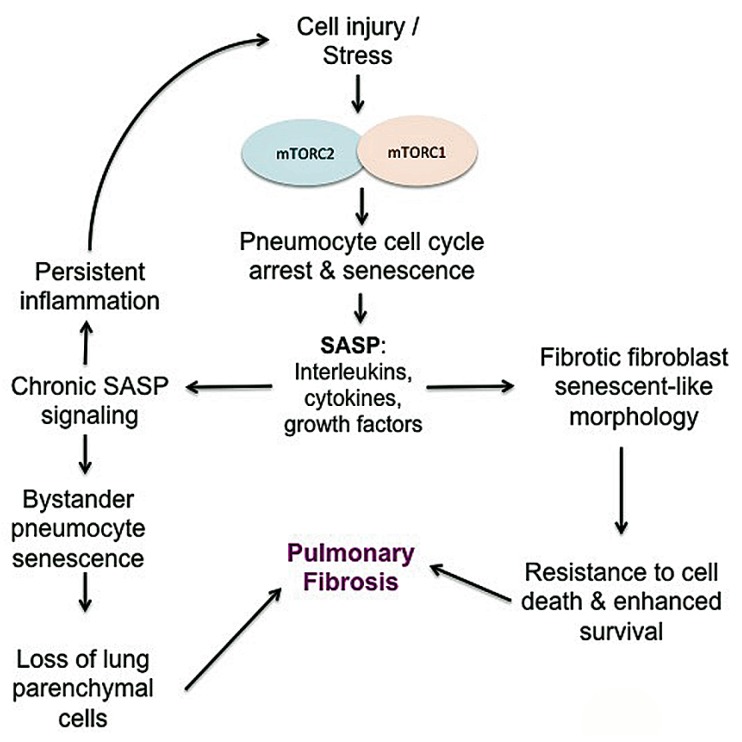
Proposed interaction between mTOR and the induction of SASP signals. Senescent pneumocytes may contribute to pro-inflammatory and pro-fibrotic signals, which further contribute to the development of pulmonary fibrosis through the loss of parenchymal cells and selection for improved fibroblast survival.

The role of stress-induced senescence in RIPF has received recent attention. Senescence is an irreversible cell growth arrest that can occur in several cell types in response to aging or following significant cellular damage. Stress-induced senescence can result from DNA damage following radiation or other insults that create ROS. In a murine model of radiation-induced fibrosis, senescence of type II pneumocytes occurred in a time and dose-dependent manner following radiation, reducing the stem cell compartment [73]. The lack of type II pneumocytes is sufficient in mice to induce RIPF and the recognition that pneumocytes are sensitive to both radiation-induced apoptosis and senescence has important consequences [73,225]. A complex mix of pro-inflammatory, immunomodulatory, angiogenic, and mitogenic cytokines comprise the secretory profile of senescence (SASP) [72,226]. These SASP cytokines include IL-6, IL-1, vascular endothelial growth factor (VEGF), epidermal growth factor receptor (EGFR), and matrix metalloproteinases (MMPs), which have been implicated in radiation-induced fibrosis and many of which are associated with mTOR pathways (Figure 10) [73,226]. Importantly, one study suggested that irradiated senescent stem cells propagate SASP and can induce senescence in surrounding unirradiated cells [73]. This highlights the potential impact that irradiated cells can have on the adjacent microenvironment. Inhibition of mTORC1 by rapamycin in a murine radiation lung injury model demonstrated decreased SASP cytokines and type II pneumocyte senescence, resulting in an overall decrease in pulmonary fibrosis [72]. Like RIPF, cellular senescence is likely linked to IPF, suggesting similar mechanisms are at play. IPF is associated with advanced age and senescence is linked to the pathogenesis of several aging-related diseases including IPF [227]. Accelerated senescence in epithelial cells is thought to play a role in the development of IPF, as it supports the depletion of the parenchymal epithelial cells and encourages myofibroblast differentiation [228]. In one study, primary lung fibroblasts derived from IPF patients demonstrated accelerated replicative senescence, enhanced resistance to oxidative stress-induced cytotoxicity and a senescent-like morphology [229], although it is unclear exactly how mTOR alteration is associated with IPF fibroblast senescence. Lending credence to the involvement of mTOR in chronic lung disease, a recent study supports that mTOR activation drives pneumocyte senescence and contributes to inflammation in a mouse model of chronic obstructive pulmonary disease [230].

## 4. Therapeutic Targeting of mTOR in Pulmonary Fibrosis

Radiation therapy is used as a curative treatment in an increasing number of patients worldwide. In 2012 alone, an estimated 7 million cancer patients were treated with radiation therapy and overall, the number of cancer survivors continues to increase in part due to more effective therapeutic strategies [9,46,231]. As the use of radiation therapy and survivorship grow with time, more significance and attention will be placed on quality-of-life measures to ensure that adverse events associated with cancer treatment are avoided, mitigated, or effectively managed. Radiation-induced late toxicities such as fibrosis have traditionally been deemed as irreversible tissue changes, however there is some evidence that late changes may be moderately reversible [232,233,234,235]. Along with therapeutic radiation, the ability to mitigate or treat radiation fibrosis in lung and other sensitive normal tissue has tremendous implications for radiation exposure via a nuclear incident.

**Figure 11:**
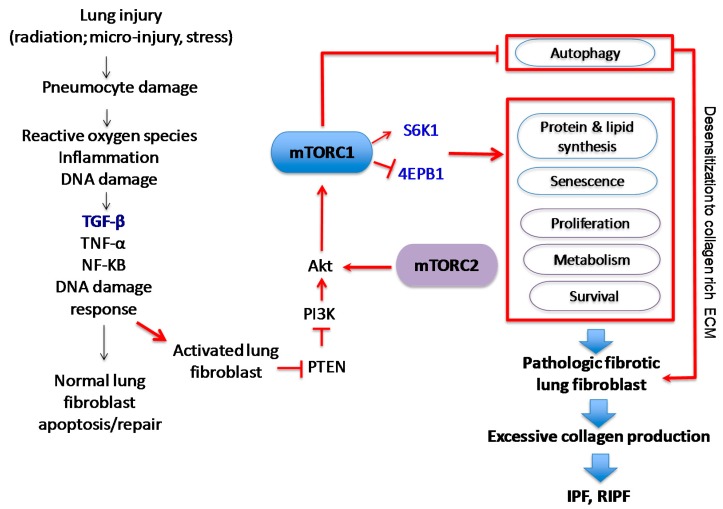
The role of mTORC1 and mTORC2 in the development and progression of idiopathic pulmonary fibrosis (IPF) and radiation-induced pulmonary fibrosis (RIPF). mTORC1 and mTORC2 are intricately involved in promoting mechanisms that favor activated lung fibroblast survival and relentless collagen production. mTORC1 inhibitors have been shown to modulate RIPF and bleomycin-induced pulmonary fibrosis, suggesting they may be effective mitigators in fibroproliferative lung disease. The arrows indicate positive regulation while T indicates the inhibition of the target molecule.

Identifying mTOR and the PI3K/AKT/mTOR pathway as a potential therapeutic target in RIPF is attractive as it is already a pathway being exploited as a radiation sensitizer in many cancers. There are thus several mTORC1, dual mTOR inhibitors and mixed mTOR/PI3K inhibitors in development or in clinical trials already that may be useful to minimize clinical pulmonary fibrosis [71,84,123]. There is solid rationale to inhibit mTOR in pulmonary fibrosis, whether it is radiation-induced, drug-induced or idiopathic in nature (Figure 11). Histologic analysis of lung tissue from IPF patients demonstrated increased mTOR expression, correlating with the degree of fibrosis and pulmonary function [236]. In both radiation-induced and bleomycin-induced lung fibrosis in mouse models, inhibition of mTORC1 appears to influence the development of fibrosis, suggesting a similar mechanistic role for the “master regulator” in pulmonary fibrosis [72,237]. Notably, when low-dose rapamycin was administered during and after fractionated radiation therapy in a mouse model, the mice receiving rapamycin had a significantly longer median survival time than the mice that received radiation alone [72]. Histologically, there were fewer fibrotic foci, decreased hydroxyproline levels, diminished inflammatory cell infiltrates, and reduced type II pneumocyte senescence in irradiated tissue from rapamycin-treated mice compared to the control lung tissue [72]. There is some clinical concern with the use of mTOR or dual mTOR/PI3K inhibitors as non-infectious pneumonitis is a well-recognized serious adverse event that can occur [238,239]. The underlying pathogenesis of mTOR-induced pneumonitis is unknown and it is generally considered reversible and dose-dependent [240,241]. The adverse event profile undermines the importance of developing and utilizing mTOR inhibitors for prevention, mitigation, or treatment imperative [46]. Interestingly, two recent clinical studies investigating the use of mTORC1 inhibitors (temsirolimus, everolimus) in patients with primary or metastatic renal cell carcinoma associated the development of drug-induced pneumonitis with improved outcome, effectively doubling the survival time compared to patients that did not develop pneumonitis [240,242]. Research such as this highlights how different dosing strategies may be essential depending on the purpose. In the murine study that demonstrated rapamycin effectively mitigated the development of RIPF, low doses of rapamycin were administered starting 2 days prior to radiation and continued for 16 weeks following fractionated radiation therapy [72]. As the authors’ pointed out, the long-term dosing used in that study was considerably lower than the high dose strategies used for radiosensitization or for tumor control [72,243,244]. The use of mTOR inhibitors will also require an understanding of the post-translational regulation of mTOR complexes that alter downstream activity; these may also affect inhibitor potency [245,246,247]. As mTORC1 contributes to the regulation of gene transcription, the elucidation of post-translational modifications in mTOR downstream targets may reveal new therapeutic targets in diseases linked to aberrant mTOR signaling [248]. It is prudent for in vitro preclinical and clinical research to address the dosing regimens and strategies most suitable to the proposed use in pulmonary fibrosis. This highlights the importance of RIPF models, as various stages of pulmonary injury and ongoing fibrosis can be fully investigated.

Given the similarities between the underlying fibroblast-driven fibrosis in IPF and in RIPF, it is therefore not surprising studies have shown that mTOR inhibition is also effective at suppressing the fibrotic process. In one study, a novel and potent dual mTOR/PI3K inhibitor (GSK2126458) that completed phase I oncology trials was capable of inhibiting PI3K signaling and functional response in IPF fibroblasts derived from patients with fibrotic foci [249]. In another study, a dual mTORC1 and mTORC2 inhibitor (MLN0128) exhibited potent anti-fibrotic activity in both in vitro and in vivo models [95]. As several dual mTOR and mTOR/PI3K inhibitors are currently under evaluation for use several solid cancers, these studies may also inform development of effective drugs for use against pathologic pro-fibrotic fibroblasts that perpetuate fibrosis. As the role of TGF-β is central to the fibrotic process and is linked to both mTORC1 and mTORC2, a rational therapeutic consideration may be the dual inhibition of mTOR and TGF-β. mTOR inhibition may alter PI3K signaling in pathological fibroblasts while TGF-β inhibition may minimize AKT activation and EMT, therefore decreasing a continued source for (myo)fibroblasts. Because TGF-β is a ubiquitous cytokine that is important in normal tissue homeostasis, selective TGF-β inhibition or localized/aerosolized drug delivery methods that target fibrotic fibroblasts may prove useful in combination with mTOR inhibitors [250,251,252]. Pirfenidone and nintedanib are both FDA approved for use in IPF with promising safety profiles [12,253,254,255]. Although the mechanism of action of pirfenidone is not completely understood, the antifibrotic effects are thought to occur indirectly through decreased TGF-β signaling, PI3K/AKT inhibition, and reduced mitochondrial ROS production, and decreased EMT [256,257,258,259]. Nintedanib is a tyrosine kinase inhibitor that blocks platelet-derived growth factor receptor, fibroblast growth factor receptor and Src (among others), all of which have been shown to be important in EMT and fibroblast proliferation, migration and differentiation [255,260]. Combining rational targeted strategies may reveal optimal methods in which to prevent, mitigate, or treat pulmonary fibrosis, regardless of the underlying insult. Thoughtful and strategic studies will be needed, however, to identify optimal dosing and combination approaches. For example, prevention of RIPF may occur with a drug regimen different than that for the mitigation of early stage IPF. This once again underscores the importance of developing relevant model systems in which to effectively study altered interventional strategies.

## 5. Concluding Remarks

It is clear that mTOR plays a central role in sensing environmental stress and regulating metabolic pathways that influence lung fibroblast growth and survival. As the function and regulation of mTOR is studied and understood across various cell types, our understanding of how mTOR regulates pneumocytes and lung fibroblasts and repair from various lung injuries will help determine the role of mTOR inhibitors in modulating fibrosis. As we have presented in this review, the actions of mTOR are far-reaching and significantly affect cell growth, proliferation, survival, inflammatory signals, EMT, metabolism, senescence, and autophagy. Given the similarities in the underlying fibrotic process, anti-fibrotic approaches studied in IPF or RIPF through mTOR modulation is likely to directly influence progression of both disease entities. The notion that several mTOR inhibitors are capable of mitigating both IPF and RIPF is encouraging and present novel therapeutic opportunities.
